# Males and Females Contribute Unequally to Offspring Genetic Diversity in the Polygynandrous Mating System of Wild Boar

**DOI:** 10.1371/journal.pone.0115394

**Published:** 2014-12-26

**Authors:** Javier Pérez-González, Vânia Costa, Pedro Santos, Jon Slate, Juan Carranza, Pedro Fernández-Llario, Attila Zsolnai, Nuno M. Monteiro, István Anton, József Buzgó, Gyula Varga, Albano Beja-Pereira

**Affiliations:** 1 Ungulate Research Unit, Cátedra de Recursos Cinegéticos y Piscícolas (CRCP), University of Córdoba, Córdoba, Spain; 2 Department of Animal and Plant Sciences, University of Sheffield, Sheffield, United Kingdom; 3 Biology and Ethology Unit, University of Extremadura, Cáceres, Spain; 4 Guardería Rural, Mancomunidad Integral de Municipios Centro (MIMC), Calamonte, Spain; 5 Centro de Investigação em Biodiversidade e Recursos Genéticos, Universidade do Porto (CIBIO-UP), Vairão, Portugal; 6 Instituto de Ciências Agrárias e Ambientais Mediterrânicas (ICAAM), Universidade de Évora, Évora, Portugal; 7 Innovación en Gestión y Conservación de Ungulados S.L., Cáceres, Spain; 8 Research Institute for Animal Breeding and Nutrition, Herceghalom, Hungary; 9 University of Kaposvár, Kaposvár, Hungary; 10 Centro de Investigação em Biomedicina (CEBIMED), Faculty of Health Sciences, University Fernando Pessoa, Porto, Portugal; 11 Forest Management and Wood Industry Share Company (SEFAG, Somogyi Erdő- és Fafeldolgozó Gazdaság), Kaposvár, Hungary; German Primate Centre, Germany

## Abstract

The maintenance of genetic diversity across generations depends on both the number of reproducing males and females. Variance in reproductive success, multiple paternity and litter size can all affect the relative contributions of male and female parents to genetic variation of progeny. The mating system of the wild boar (*Sus scrofa*) has been described as polygynous, although evidence of multiple paternity in litters has been found. Using 14 microsatellite markers, we evaluated the contribution of males and females to genetic variation in the next generation in independent wild boar populations from the Iberian Peninsula and Hungary. Genetic contributions of males and females were obtained by distinguishing the paternal and maternal genetic component inherited by the progeny. We found that the paternally inherited genetic component of progeny was more diverse than the maternally inherited component. Simulations showed that this finding might be due to a sampling bias. However, after controlling for the bias by fitting both the genetic diversity in the adult population and the number of reproductive individuals in the models, paternally inherited genotypes remained more diverse than those inherited maternally. Our results suggest new insights into how promiscuous mating systems can help maintain genetic variation.

## Introduction

The maintenance of genetic diversity in populations is strongly influenced by mating systems, which ultimately determine how variation is transmitted across generations [1], [2]. To help maintain diversity, both sexes should transmit a high proportion of the genetic diversity they contain; this is most likely under a scenario where a high proportion of males and females reproduce, with both sexes showing a low variance in reproductive success [3]. However, some mating systems might reduce the amount of genetic diversity transmitted by males or females.

Mating systems have been traditionally classified as monogamous, polygynous, polyandrous and promiscuous or polygynandrous [4], [5]. Under monogamy, each sexually mature individual within the population mates with only one individual of the opposing sex. Contrastingly, under polygynandry males and females can have multiple mates. Intermediate between these two extremes are polygyny, where males can mate with multiple females, and polyandry, where females can mate with multiple males. Each mating system type is thus associated with a sex-specific number of reproductive individuals or sex-specific variance in reproductive success, so the relative contribution of males and females to genetic diversity in the next generation tends to differ [1]–[3].

The amount of genetic diversity depends on the effective size (*N_e_*) of populations, which can be defined as the size of an ideal population, with constant finite size and random union of gametes, and where the amount of change in allele frequencies due to genetic drift is the same as the actual population under study [6]. In populations of constant size, the rate of genetic drift is mainly determined by variance in reproductive success and the sex ratio of breeding adults, which are both properties that can define a species’ mating system. For instance, since polygyny increases variance in male reproductive success, it tends to reduce genetic diversity due to a negative effect on *N_e_* [7]–[11].

The production of multiple offspring by a female in a single brood might also play an important role in determining *N_e_* and, hence, in the maintenance of genetic diversity in populations. If a single male sires all of the offspring in a brood (single paternity), *N_e_* is reduced, because of a high variance in male reproductive success, especially in highly polygynous species [7], [12]. Variance in reproductive success could be further increased if there are differences in survival rate between litters. A reduction in *N_e_* may be partially offset if more than one male sires the offspring within a litter (multiple paternity; *MP*). Traditionally, it has been accepted that *MP* reduces the variance in male mating success, increases *N_e_* and helps maintain genetic diversity [12]–[14]. However, there is an ongoing debate about the effects of *MP* on genetic diversity and *N_e_*. For instance, it has been proposed that *MP* might decrease *N_e_* and genetic diversity because *MP* might actually increase the variance in male reproductive success when compared to serial monogamy (the most common form of monogamy in which females switch partners between seasons) [15]. Ultimately, the effect of *MP* on *N_e_* is likely to depend on population parameters such as the distribution of within-litter paternity [16].

When assessing sex-specific contributions to genetic diversity, the use of a limited number of samples instead of an entire population can cause an important sampling bias. For instance, in polygynous systems it is expected that females contribute more genetic diversity than males because of the low number of reproductive males and the high variance of male reproductive success [7], [8]. However, this conclusion might only be visible when all (or a substantial part of) the population is analysed. Otherwise, a small sample of a polygynous population might capture only a limited group of females that coincidentally reproduced with different males. In this case a system in which males and females transmitted similar amounts of genetic diversity to the next generation could be described. To take into account sampling bias, studies on sex-specific contributions to genetic diversity need to create a framework where analyses of empirical data can be interpreted in light of the effect of different sampling regimes.

The mating system of the wild boar (*Sus scrofa*) has been traditionally described as polygynous based on the spatial distribution of reproductive individuals and on the degree of sexual dimorphism [17]. Females constitute family groups and males only join female groups during the rutting period [17], [18]. In the rutting season adult males try to monopolyze females and pre-copulatory male-male mate competition has driven the evolution of structures and behaviours used in aggressive fights [19], [20]. Different capacity of males to monopolize female groups has been observed depending on the studied population [17], [21]. Although males can monopolize entire female groups [21], the highly synchronized receptivity might reduce the potential for polygyny [4], [17], [22].

On the other hand, observations of the social organization of the species, of large testicles relative to body size, and the fact that females reproduce by litters, have led several authors to propose the existence of *MP* [17], [23]. The wild boar is a highly polytocous (many offspring per birth) species in which litter size is distributed around a mean of ∼4.6 [24]. Recently, relatively high rates of *MP* in wild boar populations have been reported [25], an observation that might shift the description of the species mating system, from polygynous to polygynandrous or promiscuous.

Dispersal in the wild boar is male biased [17]. Females in a given area are normally philopatric and they drive out their sons from family groups in the mating season after the birth [26]. After the expulsion, males show high dispersal rates and large ranges. Sex-biased dispersal has consequences on the genetic structure of populations and it might produce differences in the genetic diversity of males and females at breeding areas [7]. Under a male biased dispersal pattern a higher genetic diversity is expected in the subpopulation of males.

In wild boar populations, female monopolyzation might decrease the genetic diversity transmitted by males to the following generation [7]–[11]. Contrarily, *MP* might increase the contribution of males to the genetic diversity [12]–[14]. In the promiscuous mating system of the wild boar, the relative contribution of males and females to the genetic diversity of the following generation can not be easily predicted. The knowledge of each sex relative contribution to the next generation is important to understand the evolutionary implications of a particular mating system.

In this study, we investigated the relative contribution of reproducing males and females to the genetic diversity of their progeny in different wild boar populations. We determined which alleles were transmitted to progeny by both fathers and mothers to measure sex-specific contributions to diversity. Initially this was done without controlling for any further factors. Subsequently, we used genetic structure analyses to establish the actual number of populations, before measuring the contribution of males and females to genetic diversity while controlling for both the amount of genetic variation in the adult population and the number of reproductive individuals. We used simulations to assess the impact of possible sampling biases.

## Materials and Methods

### Populations and Sample collection

The samples originated from either the Iberian Peninsula or Hungary (Figures A and B in S1 File). We collected tissue samples from the carcasses of specimens legally culled by hunters in officially organized hunting events. This study was carried out in strict accordance with the law. No animals were killed specifically for this study. Hunters used rifles of different calibres depending on user preference. Iberian Peninsula samples were obtained in five hunting events conducted in Évora (38°34′02″N, 7°54′32″O), Alqueva (38°12′55″N, 7°31′59″O), Vila Viçosa (38°46′34″N, 7°25′02″O), Azagala (39°11′11″N, 6°50′28″O) and Santa Amalia (39°43′28″N, 5°57′13″O). In this region, hunting is characterized by the use of packs of dogs that are released within forest areas to move the wild boar outwards to the sites where hunters are placed. In Hungary, culled individuals were collected in nine hunting events conducted in Kisbajom (46°17′42″N, 17°29′11″E), Lábod (46°13′17″N, 17°30′7″E), Szulok (46°2′18″N, 17°30′19″E), Cserénfa (46°17′50″N, 17°51′12″E), Kereki (46°47′18″N, 17°53′34″E), Kereki Kapasi (46°45′24″N, 17°55′49″E), Pusztaszemes (46°45′53″N, 17°55′55″E), Karád (46°45′24″N, 17°56′7″E) and Tótokilap (46°49′29″N, 17°57′10″E). Here, hunting is characterized by the formation of a long line of people who move the wild boar towards the hunter sites. All samples were collected from hunting events that took place between November 2008 and February 2009. Around 80% of females were pregnant at the moment of capture (P. Fernández-Llario, unpublished data).

Tissue samples were collected from a random sample of 91 males, 110 pregnant females (mothers) and 502 foetuses (see S1 and S2 Tables for additional details on the distribution of samples). Pregnant females were only used if their foetuses were sufficiently developed to obtain a tissue sample. We recorded the foetuses belonging to the same litter and the mother of each litter. Male samples were collected from males older than 1 year to ensure that they had already been expelled from the family groups. Age of males was estimated using the body size or the chronology of teeth eruption [26]. Adult samples consisted of pieces of ear cartilage and a piece of muscle was collected from foetuses. Samples were placed in a 1.5 ml eppendorf tube and preserved in 96% ethanol. The samples were extracted with JETQUICK Tissue DNA Spin Kit (Genomed, Löhne) according to the manufacturer’s instructions.

We did not conduct refined methods for the estimation of population sizes. However, it is certain that entire populations were not sampled; past experience with the study areas suggests a sampling intensity between 10–20%. For instance, in the Iberian area of Azagala an approximate number of 200 females were inferred before the hunting event (P. Fernández-Llario, unpublished data). In Azagala we collected 35 females, approximately 18% of females in the area. In the Iberian area of Santa Amalia there were around 100 females (P. Fernández-Llario, unpublished data); 13 females were sampled giving a sampling intensity of ∼13%.

### Microsatellite genotyping

All 703 samples used in this study were genotyped for a set of 14 microsatellite markers designed for parentage analyses in wild boar (Sw24, S0155, Sw936, Sw2410, S0005, Sw632, Sw857, S0226, Sw72, Sw240, S0068, S0101, Sw122, Sw2008) [27]. We performed two multiplex polymerase chain reactions with a total volume of 10 µl containing 10 ng of genomic DNA, 10 mM of primer mix, Qiagen Multiplex PCR Master Mix (QIAGEN, Hilden) and water [27]. The multiplex products were added to a mix of denaturant formamide and size standard (Gene Scan 500 LIZ size standard) and run on a 3130 XL Genetic Analyzer (Applied Biosystems) sequencer. GENE MAPPER v4.0 (Applied Biosystems, USA) was used for allele calling (see S3 Table for details about microsatellite markers).

### Analyses of wild boar data

We did not have previous in-depth knowledge of the number and distribution of populations within the study areas. Therefore, STRUCTURE 2.0 [28] was used to delineate clusters of individuals on the basis of their genotypes at multiple loci. Accordingly, the STRUCTURE approach was implemented in adult males and pregnant females (201 individuals). To determine the number of genetic clusters (*K*), ten independent runs from *K* = 1 to *K* = 8 were carried out with 500,000 iterations, following a burn-in period of 100,000 iterations. As an additional assessment, we conducted Structure analyses (10 independent runs, 500,000 iterations, 100,000 burn-in, from *K* = 1 to *K* = 5) for the Iberian Peninsula and Hungary populations separately, to look for minor genetic clustering within the major geographical areas. We regarded genetic clusters as populations if all the hunting locations of the clusters belonged to the same geographic neighbourhood.

Genetic diversity was measured using Shannon’s index [1], [11]:
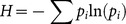
where *p_i_* is the frequency of the *i* allele in the genotype set. This index takes into account the number of different alleles and the frequency of each allele.

For each population and marker four sex-specific genetic diversity values were obtained: genetic diversity in the random sample of adult males (diploid individuals), genetic diversity in mothers (also diploids), diversity in the paternally transmitted genetic component of progeny (haploid paternal genotype) and diversity in the maternally transmitted genetic component of progeny (haploid maternal genotype). Paternal and maternal genotypes of the progeny (haploid parental genotypes) were constructed by comparing each foetus’ genotype with its mother’s genotype [11], [29] and assigning the non-maternal allele to the father. Ambiguities (foetus and mother shared the same heterozygous genotype) [30] were dealt with by following three different methods:

- Method 1. Ambiguities were removed and parental genotype diversity was quantified using only those alleles where there was no doubt about whether they were paternally or maternally inherited.
- Method 2. Alleles were assigned to the paternal genotype at random but with a probability proportional to the relative frequency of the ambiguous alleles in the population the foetus belongs to. In this case, the probability of assigning the most common allele to the paternal genotype increased.
- Method 3. Alleles were assigned to the maternal genotype at random but with a probability proportional to the relative frequency of the ambiguous alleles in the population. In this case, the most common allele was more likely to be assigned to the maternal genotype.

In Method 1, only a single genetic diversity value for paternal and maternal genotypes in each population and marker was obtained. For Methods 2 and 3, a 1,000 replication process was conducted for the entire data set, which reproduced a mean genetic diversity for paternal and maternal genotypes in each population at each marker.

We quantified the following mating system characteristics of the population samples we studied: number of fathers and mothers, mean number of mates per mother (*MP* rate), mean reproductive success for fathers and mothers, and variance of father and mother reproductive success. The number of mothers, as well as the mean and variance of mother reproductive success were quantified using the samples of pregnant females and the distribution of the observed litter sizes. The remaining mating system parameters were inferred using the COLONY 2.0 software [31]. This software uses a group-likelihood approach to infer sibship and parentage among individuals. COLONY allows both sexes to be polygamous and it can accommodate genotyping errors. The genotypes of mothers, offspring and the random sample of males, as well as the known mother-offspring relationships were included in the input files. The rate of allelic dropout was set to 0.01 and the rate of other kind of genotyping errors, including mutations was set at 0.005 [32]. An analysis using 0.0001 as the genotyping error rate did not change the main results (data not shown). To obtain the number of fathers, as well as the mean and variance of father reproductive success, the distribution of the number and size of paternal families was calculated from the configurations with the highest likelihood values.

The number of fathers and mothers was also used as a covariate to account for the sampling bias in the comparison in genetic diversity between paternal and maternal genotypes (see below). The comparison between paternal and maternal contributions to genetic diversity after controlling for covariates such as the number of parents is the main focus of this work, so we additionally inferred the number of fathers by using an alternative paternity inference software: MOL_COANC [33]. This software uses the “blind search algorithm” that finds the genealogy yielding the coancestry matrix with the highest correlation with the molecular co-ancestry matrix, calculated using the markers. We used the estimated genealogy obtained with MOL_COANC to calculate the number of fathers in the populations.

The data were analysed fitting Linear Mixed Models (LMMs) in a Bayesian framework, using Markov chain Monte Carlo (MCMC) techniques. Firstly, genetic diversity estimates in adult males and mothers were compared by conducting a LMM with sex-specific adult genetic diversity as the dependent variable, sex as fixed factor, and marker and population as random effects (N = 112∶14 markers * 4 populations * 2 sexes). Secondly, we compared the amount of genetic diversity contributed by males and females to the following generation by performing a LMM with sex-specific genetic diversity in parental genotypes (haploid genetic diversity) as the dependent variable, sex (paternal *versus* maternal genotypes) as fixed factor, and marker and population as random factors (N = 112∶14 markers * 4 populations * 2 parental genotypes). Finally, genetic diversity contributed by males and females was compared after controlling both for the sex-specific genetic diversity in adults and the number of fathers and mothers. In this case, a LMM was performed with sex-specific genetic diversity in parental genotypes as the dependent variable, sex-specific genetic diversity in adults (in the random sample of males for paternal genotype and in the mothers for maternal genotype), number of parents in the population (fathers for paternal genotype and mothers for maternal genotype) and sex as fixed factors, and marker as a random effect (N = 112∶14 markers * 4 populations * 2 parental genotypes; see information associated with S1 Fig. for an assessment regarding this analysis). We repeated the analyses with genetic diversity estimates obtained by each of the three methods for dealing with ambiguous alleles (see above). All statistical analyses were conducted using the *MCMCglmm* package [34] in R [35]. We show results under chain convergence and independence of consecutive iteration values. To determine the existence of systematic changes as a function of iteration number, we conducted autocorrelation analyses. As autocorrelation between successive iteration values was small, we assume that independent samples from the posterior were obtained. Model selection was made using the deviance information criterion.

### Simulation analyses

Because estimates of genetic diversity transmitted to the following generation by males and females might be influenced by sampling biases (see above), we simulated a promiscuous population and assessed the effect of sample size and sampling intensity on the relative contribution of males and females to genetic diversity. By simulating different scenarios it was possible to interpret our empirical results in the context of knowledge of whether incomplete sampling caused bias in downstream analyses. In the simulations, we assessed the number of inferred reproductive males and the relative contribution of males and females to genetic diversity when the number of sampled females was varied. The following parameters were explored in the simulations: the variance in male reproductive success (*V_males_*), the *MP* rate, the mean litter size (*LS*) and the variance in female reproductive success (*V_females_*).

We used information in our wild boar populations to simulate a realistic initial population in which we modelled the sampling bias. In this initial population we used 110 females in which the litter size distribution was the same as that in the studied wild boar females. Mean litter size (*LS*) was 4.564 and variance in litter size (*V_females_*) was 3.367. We incorporated 110 males to this simulated population. For each individual in the population, genotypes were randomly assigned for a locus with 6 alleles (the mean number of alleles we found in our wild boar populations was 6.21). Females were allowed to randomly mate with males. To simulate *MP*, females mated with two males (*a* and *b*). Within litters, both males were assumed to have equal probability of paternity. When the litter size was odd, the number of offspring sired by male *a* was higher. To simulate polygyny, males were allowed to mate with more than one female. Variable male reproductive success was simulated by assuming 80% of males have a probability *p* of mating, 10% of males have a probability 2*p* of mating and 10% of males have a probability 3*p* of mating. After 1,000 replicates of this mating system, the resulting variance in male reproductive success was 15.547±2.983 (mean ± SD). For the simulated population one female was randomly sampled and the number of males she mated with was recorded, along with, the genetic diversity of the maternally and paternally inherited genotypes. We also quantified the difference between genetic diversity of maternally and paternally-inherited genotypes. Then, the same values were recorded after randomly sampling 2 to 110 females. Thus, we recorded the mean number of mates (reproductive males) and the genetic diversity of the maternally and paternally inherited genotypes for sampling regimes that ranged from 1 female to the entire subpopulation of females. We repeated each scenario 1,000 times.

Following the initial simulations, parameters of interest (*V_males_*, *MP*, *LS* and *V_females_*) were modified and the process was repeated. For each parameter two additional scenarios were created; one below and one above the initial condition. For *V_males_* we used the same parameters as the initial population but with the following *V_males_* values: all males have equal mating success (yielding a value of *V_males_* = 10.230±1.613; after 1,000 simulations); 80% of males have a probability *p* of mating, 10% of males have a probability 2*p* of mating and 10% of males have a probability 5*p* of mating (yielding *V_males_* = 25.757±5.441; after 1,000 simulations). For *MP*, two additional scenarios were simulated: females reproduced with one (single paternity) or with three males (*a*, *b* and *c*). Within litters, all males were modelled to have an equal number of paternities. When the litter size was not a multiple of 3, the number of offspring sired was *a*≥*b*≥*c*. For *LS* the following scenarios were explored: for all females we decreased the litter size by 2 offspring (yielding *LS* = 2.718; the females producing 1 or 2 offspring in the initial population were assumed to produce just 1 offspring in this situation); for all females we increased the litter size by 2 offspring (yielding *LS* = 6.564). Finally, for *V_females_* the two scenarios were: for females with more than 4 offspring in the initial population we decreased the litter size by 1 offspring and for females with less than 5 offspring we increased the litter size by 1 offspring (resulting in *V_females_* = 1.449); for females with more than 4 offspring in the initial population we increased the litter size by 1 offspring and for females with less than 5 offspring we decreased the litter size by 1 offspring (resulting in *V_females_* = 7.077; the females producing 1 offspring in the initial population were assumed to also produce 1 offspring in this situation). Therefore, 8 additional scenarios were simulated. The impact of incomplete sampling on estimates of paternal and maternal contributions to offspring diversity was examined using the same procedure as for the initial population.

The values of the mating system parameters used in simulations for the initial and additional populations were similar to those found in the sampled wild boar population.

## Results

### Genetic structure and mating system in adult populations

**Figure 1:**
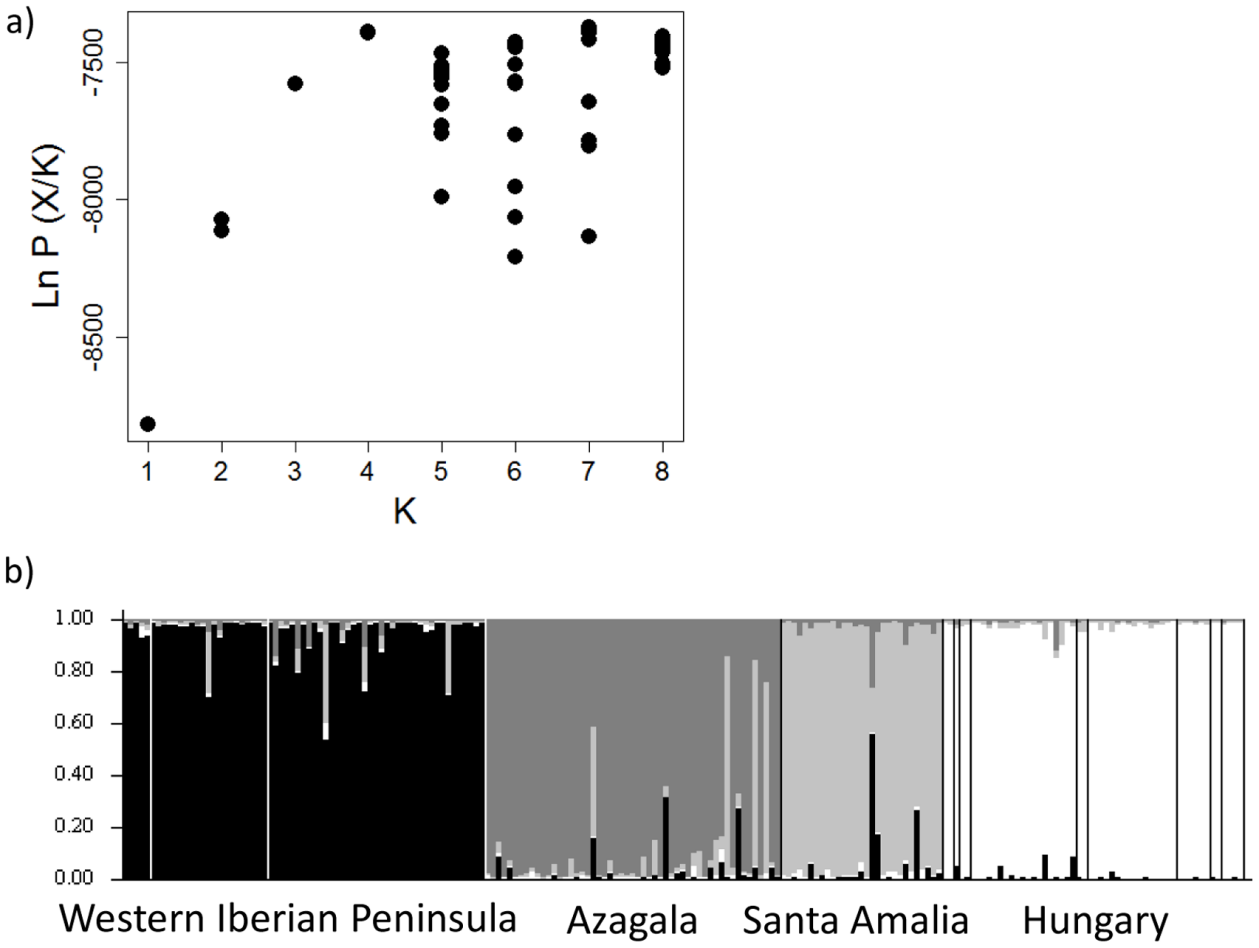
Structure software results. a) Log-likelihood values (ten independent runs) for each assessed *K* value. b) Membership coefficient of probability for *K* = 4. Each individual is represented by a thin column which is portioned into four segments with different grey intensity depending on the individual’s estimated membership fraction (Y axis) in *K* cluster. Vertical white or black lines divide individuals sampled at different hunting events. Hunting events are located from west to east: Évora, Alqueva, Vila Viçosa, Azagala, Santa Amalia, Kisbajom, Lábod, Szulok, Cserénfa, Kereki, Kereki Kapasi, Pusztaszemes, Karád and Tótokilap.

STRUCTURE grouped the individuals into four genetic clusters (*K* = 4; Fig. 1a). Despite the existence of some immigrants (animals from other genetically different populations) or descendants of immigrant individuals, the genetic clusters closely matched four geographic areas: western Iberian Peninsula (WIP; grouping 3 hunting locations), Azagala (AZA; one hunting location in the Iberian Peninsula), Santa Amalia (SAM; one hunting location in Iberian Peninsula) and Hungary (HUN; grouping 9 hunting locations) (Fig. 1b). After conducting the STRUCTURE analyses separately for Iberian Peninsula and Hungary, the same genetic clusters were obtained for the Iberian Peninsula (Hungary was a unique separate cluster) (see Fig. S2a, S2b and S2c in S2 File).

The mating system of each wild boar population is summarised in Table 1. In three out of the four populations studied, the number of fathers estimated with COLONY was greater than the number of mothers. *MP* was found in all populations and the average number of mates per mother (*MP* rate) was 1.781. In all populations the coefficient of variation (CV = standard deviation/mean) of reproductive success was higher in fathers than in mothers (WIP: CV = 0.741 for fathers, CV = 0.322 for mothers; AZA: CV = 0.608 for fathers, CV = 0.402 for mothers; SAM: CV = 0.625 for fathers, CV = 0.291 for mothers; HUN: CV = 0.944 for fathers, CV = 0.316 for mothers; results from Table 1). As an additional result, there was a strong positive relationship between mean litter size and the variance in father reproductive success (*N* = 4; Pearson’s *r* = 0.994, *p*<0.01; Spearman’s *rho* = 1, *p* = 0.083).

**Table 1:** Mating characteristics of populations.

	N_mothers_	N_fathers_	MP rate	M_mothers_	M_fathers_	V_mothers_	V_fathers_
WIP	27	30.8	1.8	4.3	3.8	1.9	7.8
AZA	35	34.3	1.7	3.9	3.9	2.4	5.8
SAM	13	17.8	1.7	3.5	2.5	1.0	2.5
HUN	35	38.2	1.9	5.9	5.4	3.5	26.0

N_mothers_: number of sampled mothers. N_fathers_: number of inferred fathers. MP rate: multiple paternity rate. M_mothers_: mean litter size. M_fathers_: mean reproductive success of fathers. V_mothers_: variance in litter size. V_fathers_: variance in father reproductive success. Data for mothers were directly estimated from the sampled pregnant females. Data for fathers were estimated using Colony analyses of maternal and foetus genotypes.

Genetic diversity was similar in adult males and pregnant females in all populations (LMM fitted using MCMC: posterior estimate of the effect = −0.005, 95% credible interval = −0.113/0.113, *p*_MCMC_ = 0.910).

### Contribution of males and females to offspring genetic diversity

**Figure 2:**
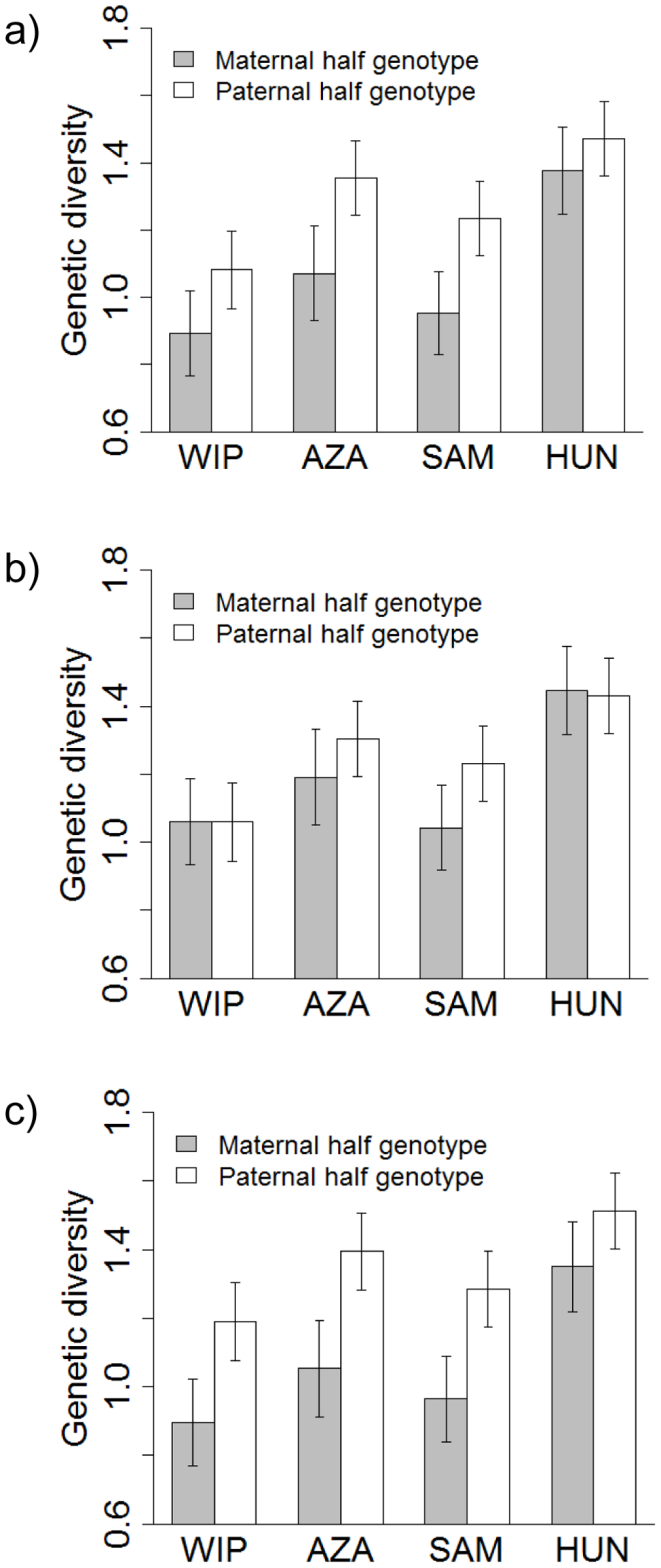
The contribution of males and females to offspring genetic diversity. Paternal genotypes inference performed by: a) Method 1; b) Method 2; c) Method 3. Results are shown separately for each population. WIP: Western Iberian Peninsula; AZA: Azagala; SAM: Santa Amalia; HUN: Hungary. Figure shows means and standard errors of observed values.

When Methods 1 and 3 were used for assigning paternal and maternal genotypes, paternally inherited genotypes were more diverse than maternally inherited genotypes (Table 2; Fig. 2). When Method 2 was used there was no significant sex difference (although there was a tendency for paternally inherited genotypes to be more diverse).

**Table 2:** Fixed effects of a LMM fitted using MCMC in which we compared the genetic diversity in paternally and maternally transmitted genotypes.

a) Method 1	post. Mean	lower 95% CI	upper 95% CI	P_MCMC_
Intercept	1.076	0.692	1.427	<0.001
Sex	0.211	0.113	0.327	<0.001
**b) Method 2**	**post. Mean**	**lower 95% CI**	**upper 95% CI**	**P_MCMC_**
Intercept	1.189	0.863	1.508	0.004
Sex	0.075	−0.021	0.170	0.126
**c) Method 3**	**post. mean**	**lower 95% CI**	**upper 95% CI**	**P_MCMC_**
Intercept	1.060	0.738	1.348	0.002
Sex	0.279	0.176	0.376	<0.001

Table shows the posterior estimate of the effects, the 95% credibility interval and the probability that the null hypothesis is true (effect = 0). Parental allele assignment performed by: a) Method 1. b) Method 2. c) Method 3. Maternal genotype as reference.

Regardless of the method employed, the amount of genetic diversity in inherited genotypes was positively related to both the genetic diversity of adults, and to the number of parents (Table 3; see Fig. S3a and S3b in S3 File). However, in models that fitted these variables, males still transmitted more genetically diverse genotypes than did females to the next generation (Table 3; see Fig. 2). Results after using MOL_COANC for paternity analyses are shown in Tables A, B and C in S4 File.

**Table 3:** Fixed effects of a LMM fitted using MCMC in which we compared the genetic diversity of paternally and maternally transmitted genotypes after controlling for the genetic diversity in adults and the number of reproductive individuals.

a) Method 1	post. mean	lower 95% CI	upper 95% CI	P_MCMC_
Intercept	−0.263	−0.410	−0.111	<0.001
Genetic diversity in adults	0.960	0.883	1.045	<0.001
Number of reproductive individuals	0.006	0.002	0.011	0.006
Sex	0.198	0.126	0.270	<0.001
**b) Method 2**	**post. mean**	**lower 95% CI**	**upper 95% CI**	**P_MCMC_**
Intercept	−0.096	−0.229	0.017	0.134
Genetic diversity in adults	0.930	0.865	0.997	<0.001
Number of reproductive individuals	0.005	0.002	0.009	0.006
Sex	0.059	0.009	0.128	0.044
**c) Method 3**	**post. mean**	**lower 95% CI**	**upper 95% CI**	**P_MCMC_**
Intercept	−0.214	−0.356	−0.068	0.004
Genetic diversity in adults	0.937	0.859	1.011	<0.001
Number of reproductive individuals	0.005	0.001	0.009	0.010
Sex	0.269	0.194	0.335	<0.001

Table shows the posterior estimate of the effects, the 95% credibility interval and the probability that the null hypothesis is true (effect = 0). a) Method 1. b) Method 2. c) Method 3. When Sex factor was coded as “males” dependent variable was genetic diversity of paternal genotypes, Genetic diversity in adults referred to genetic diversity in the random sample of males and Number of reproductive individuals referred to number of inferred fathers. When Sex factor was coded as “females”, dependent variable was genetic diversity of maternal genotypes, Genetic diversity in adults referred to genetic diversity in pregnant females and Number of reproductive individuals referred to number mothers. Females were used as reference for sex factor.

### Simulations

**Figure 3:**
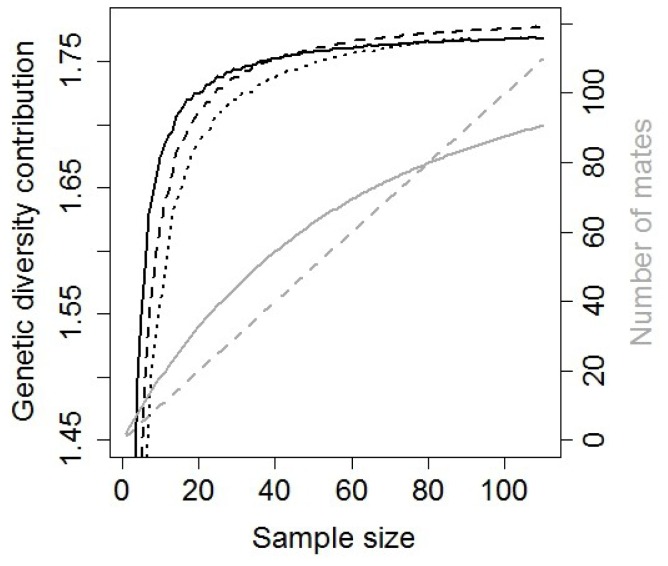
Simulation results for the initial population. Black solid line: relationship between the number of sampled females (X axis) and the diversity of paternally transmitted genotypes. Black dashed line: relationship between the number of sampled females (X axis) and the diversity of maternally transmitted genotypes. Black dotted line: relationship between the estimated number of mates (X axis) and the diversity of paternally transmitted genotypes. Grey solid line: relationship between the number of sampled females (X axis) and the estimated number of sampled reproductive males (mates; note that these values were used as X axis to construct the black dotted line). Grey dashed line: equality between the number of sampled females (X axis) and the estimated number of reproducing males.

The simulation results show an important effect of sampling intensity on the estimated relative contribution of males and females to genetic diversity (Fig. 3). If the entire population of females (110) in the initial population is sampled, the number of reproductive males is lower than the number of reproductive females, and the genetic diversity of paternally transmitted genotypes is estimated to be lower than that of maternal genotypes. However, if the proportion of females sample is lower, the pattern of estimated sex-specific differences in the number of reproducing individuals and sex-specific contributions to genetic diversity can be reversed.

When we used the inferred number of mates (grey curve in Fig. 3) as the X-axis to represent the genetic diversity of paternally transmitted genotypes, the relationship between genetic diversity of maternal and paternal genotypes was similar along the range of sample size: the genetic diversity of maternal genotypes (dashed black line in Fig. 3) was always higher than that of paternal genotypes (dotted black line in Fig. 3).

**Figure 4:**
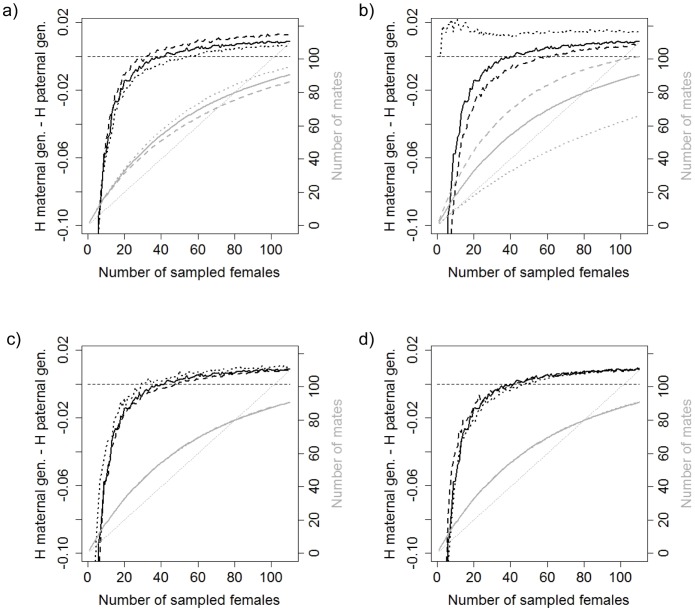
Simulation results after varying *V_males_*, *MP*, *LS* and *V_females_*. The relative diversity of maternally and paternally transmitted genotypes (genetic diversity of maternal genotypes - genetic diversity of paternal genotypes; black lines) and the estimated number of sampled reproductive males (mates; grey lines) for different female sampling intensities are shown. a) Three populations that differ in *V_males_*. b) Three populations that differ in *MP*. c) Three populations that differ in *LS*. d) Three populations that differ in *V_females_*. In all graphs, the solid line represents the initial population, the dotted line represents the *below* population (i.e. where the adjusted parameter is reduced relative to the initial conditions) and the dashed line represents the *above* population (parameter is increased relative to initial conditions). The thin horizontal black dashed line represents equal genetic diversity in maternally and paternally transmitted genotypes. The thin diagonal grey dotted line represents equality between the estimated number of reproducing males and the number of sampled females.

If *V_males_* is increased the estimates of the number of reproductive males and the relative genetic diversity of paternally transmitted genotypes become lower (Fig. 4a). However, the effect of sampling intensity was similar regardless of the value of *V_males_* (Fig. 4a). Increasing *MP* was associated with an increase in the estimated number of reproductive mates and the estimated diversity of paternal genotypes (Fig. 4b). In the simulations with *MP* set to equal 1, the number of estimated reproducing males was always lower than the number of sampled females and the estimated genetic diversity of maternal genotypes was always greater than that of paternal genotypes (Fig. 4b). The number of estimated reproductive males was not sensitive to changes in *LS* and *V_females_* (Fig. 4c and d). The increase of *LS* tended to decrease the genetic diversity of maternal genotypes relative to that of paternal genotypes (Fig. 4c). An increase in *V_females_* tended to increase the genetic diversity of maternal genotypes, but only at low sample sizes (Fig. 4d).

## Discussion

In this study, we investigated the relative contribution of males and females to genetic diversity in the following generation by sampling four genetically distinct wild boar populations. We found that the paternally transmitted genetic component was more diverse than the maternally transmitted component. After controlling for both the genetic diversity in the adult population and the number of parents in our samples, paternally inherited genotypes remained more diverse than maternally inherited genotypes.

The wild boar populations we studied displayed a promiscuous mating system in which *MP* occurs. However, the variance (and the coefficient of variation) in reproductive success was higher in fathers than in mothers in all four populations. The highest difference between father and mother variance in reproductive success was found in Hungary, where the litter size was also the highest of the study populations. Because of the higher variance of reproductive success in fathers than in mothers, a lower contribution of fathers to the genetic diversity of the following generation might be expected [7], [8]. However, we found an opposite result (see below).

The amount of genetic diversity was not different between the sampled adult males and mothers. However, we found that paternally-transmitted genotypes in foetuses were more diverse than maternally-transmitted genotypes, by two of the three methods for dealing with ambiguously inherited alleles. This result was consistent in all sampled populations (Fig. 2). The other method (Method 2) was extremely conservative, and is expected to produce estimates of greater diversity in maternal genotypes, yet there remained a trend for paternally-transmitted genotypes to be more variable.

The simulation analyses showed that paternal genotypes can be estimated to be more diverse than maternal genotypes in this system due to a sampling bias. In our wild boar populations we presumably sampled a small proportion of the population (10–20%). When sampling intensity is low, the number of sampled females is typically lower than the estimated number of mates these females mated with. As a consequence, the estimated genetic diversity of paternally-transmitted genotypes is greater than that of maternally-transmitted genotypes. Therefore, the relatively high genetic diversity of paternally-transmitted genotypes could be due to a sampling bias with little biological relevance.

The possible influence of a low sampling intensity on the comparison between maternal and paternal contributions to genetic diversity necessitated further analyses. When we compared the relationship between the number of sampled females and the genetic diversity transmitted by these females (black dashed line in Fig. 3) with the relationship between the inferred number of mates and the genetic diversity transmitted by these males (black dotted line in Fig. 3), the genetic diversity transmitted by females was always higher than that transmitted by males, regardless of sampling intensity. The difference between the amount of genetic diversity transmitted by males and females was similar under small and big sample sizes. This is because we took into account the number of reproductive males and females to compare the genetic diversity transmitted by males and females respectively. Therefore, the number of reproductive individuals was included in models to account for sampling intensity bias. If the observed difference in genetic diversity between paternal and maternal genotypes was due to the bias, this difference would be expected to disappear after fitting the number of fathers and mothers as a covariate. The genetic diversity in the random sample of males and in mothers were also included as additional covariate to compare the relative contribution of males and females to the genetic diversity of the following generation. The sex difference in genotype diversity remained after fitting the additional covariates; in fact, the difference for Method 2 now reaches statistical significance. Therefore, the observation that males transmitted more diverse genotypes than females to the following generation appears to be robust.

There are several possible explanations for why males appear to transmit more genetic diversity than females to the next generation. A non-biological explanation might be bias in the sampling procedure, if hunting would select for genetically less diverse males which are not representative of the male subpopulation. However, this is unlikely with the hunting methods used in the study populations, which have been proven to be unbiased for red deer [36]. Additionally they have been suggested as neutral hunting methods for obtaining unbiased data of wild boar populations [37].

Alternatively, males could contribute relatively greater genetic diversity if reproductive males (fathers that sired our sample of foetuses) are genetically more diverse than the overall population of reproductive-aged males (the random sample of males we used), i.e. sexual selection favoured males with the greatest genetic diversity. In this case, the inferred paternally-transmitted genotypes would not be a random sample of all male genotypes in the population. Precopulatory and postcopulatory processes might favour males with the highest genetic diversity. On one hand, male-male mate competition is a precopulatory feature of the wild boar mating system. Males have developed aggressive behaviours, weapons and defences to compete for mating opportunities [19], [20]. A low individual genetic diversity may cause males to be less well-equipped for male-male competition and therefore hinder access to reproduction [38]. If the most genetically diverse males tend to monopolize reproduction, genetic diversity contributed by males might be higher than expected, after taking into account the number of reproductive males [11]. On the other hand, poor quality ejaculates of those males with low genetic diversity [39] might also favour the success of males with the highest genetic diversity.

The relationship between heterozygosity and reproductive success has been detected in different mammal groups such as primates [40], [41]. For instance, in rhesus macaques (*Macaca mulatta*) populations, heterozygous males at MHC genes present higher reproductive success [40]. Heterozygosity at MHC genes favours the resistance to the debilitating effects of injury and parasite infections [40]. On the other hand, in mandrill (*Mandrillus sphinx*) populations, heterozygosity at microsatellite markers is associated with increased reproductive success [41]. Microsatellite markers are considered to be selectively neutral, but inbreeding depression effects or genotypic disequilibrium between single microsatellite locus and selected genes might occur [41], [42].

Finally, male-biased dispersal in wild boar populations [17] might play an important role in sex-specific contributions to next-generation genetic diversity. The fact that males disperse over longer distances than females makes them the prime promoters of genetic diversity among populations [7]. Female philopatry and high rates of male movements during the rutting period could increase the relative contribution of males to genetic diversity in the next generation at local scale [17]. However, we found that culled adult males and females did not differ in genetic diversity. Therefore, there is little evidence that sex-biased dispersal is an explanation to the observation that paternally transmitted genotypes are more diverse than maternally transmitted ones. It should be noted that sampling occurred when females were in advanced pregnancy, so estimates of male and female genetic diversity may not be representative of mating season. Data obtained by radiofrequency tagged animals (before and after rutting) coupled with DNA information obtained in the hunting period (after rutting) could help refine our findings.

The difference in genetic diversity of paternal and maternal genotypes is suggestive of sexual selection driving a high diversity in paternally-transmitted genotypes. However, we cannot conclude that males transmitted more overall genetic diversity than females because it is likely that a relatively low fraction of each entire population was sampled. The simulations showed that when a high proportion of females are sampled, the amount of genetic diversity transmitted by females exceeds that transmitted by males. However, the simulations are ‘null models’ in the sense that any process that would increase the amount of genetic diversity contributed by males was not incorporated. Sex-specific contributions to diversity in wild boar populations will depend on the balance of the processes tending to increase and decrease genetic diversity [6], [7], [11], [13].

The simulation results suggest that the existence of *MP* was the most important factor in determining whether incomplete sampling caused a sex-bias in estimates of sex-specific contributions to genetic diversity. When single-sire paternity was simulated, the bias caused by incomplete sampling lost importance (see the black dotted line in Fig. 4b). Varying all of the remaining parameters did not induce substantial changes in the sampling bias pattern. In addition the simulations showed that the relative contribution of genetic diversity by males decreased when *V_males_* increased [7], [8]. The relative contribution of genetic diversity by males increased as *MP* increased, but only when the sampling proportion was relatively low. The positive relationship between *MP* and male contribution to genetic diversity agree with both theoretical expectations [12] and empirical results [43], [44]. However, when the entire population was sampled (*N* = 110 females) the increase in *MP* did not have important consequences for estimates of the relative amounts of genetic diversity transmitted by males and females. It has been proposed that low *MP* rates have important consequences on transmission of genetic diversity only when litter sizes are large [45]. The litter sizes and the degree to which litters were sired by multiple males (real data: average *MP* = 1.781; simulations: *MP* = 1–3) might not be large enough to detect any effect of *MP* rate on estimates of sex-specific contributions to genetic diversity.

In polygynandrous and promiscuous systems multiple mating carries costs, and several non-exclusive hypotheses have been proposed to explain the evolutionary benefits of *MP* necessary for its existence [46]. One of the hypotheses is an increase of genetic diversity within litters [47], relative to single-sire litters. Increasing the genetic diversity might reduce the vulnerability of the progeny to disease, pathogen transmission and the resource competition among half-sibs [48], [49]. In addition to *MP*, polygyny-related processes such as the most genetically diverse males having greatest fitness [11], [38] or male-biased dispersal [50] might further increase the genetic diversity of litters. Polygynandrous or promiscuous mating might be considered as a system with a relatively high potential to maximise genetic diversity of litters that, in turn, produce higher quality offspring. The production of fitter offspring might be enhanced by other processes associated with polygynandry such as sperm competition and cryptic female choice [51], [52]. Polygynandry involves a wide range of evolutionary processes that tend to increase the genetic diversity across generations and to enhance the quality of descendants.

In summary, our data suggest that the promiscuous mating system of wild boar can cause male contributions to genetic diversity to exceed those of females, and that the mating system is a mechanism by which the maintenance of genetic diversity is promoted. However, care must be taken in this type of study to ensure that spurious sex-specific differences do not arise due to sampling biases.

## Supporting Information

S1 Fig **Relationship between the inferred number of reproductive individuals and the genetic diversity in paternal genotype.**
(DOC)

S1 Table **Sample sizes for each hunting site.**
(DOC)

S2 Table **Sample sizes and heterozygosity for each genetically different population.**
(DOC)

S3 Table **Genetic information of microsatellite markers.**
(DOC)

S1 File **The study area and the places in which hunting events were conducted.** This file also contains Figures A and B. Figure A, Iberian Peninsula. Figure B, Hungary.
(DOC)

S2 File **Structure results after splitting Iberian Peninsula and Hungary.** This file also contains Figure S2. Figure S2a, Log-likelihood values for Iberian Peninsula (ten independent runs). Figure S2b, Membership coefficient of probability for Iberian Peninsula (*K* = 3). Figure S2c, Log-likelihood values for Hungary (ten independent runs).
(DOC)

S3 File **Relationship between genetic diversity in parental genotypes and covariates.** This file also contains Figure S3. Figure S3a, Covariate: Number of reproductive individuals. Figure S3b, Covariate: Genetic diversity in adults.
(DOC)

S4 File **Results after using MOL_COANC for paternity analyses.** This file also contains Tables A–C. Table A, Results for Method 1. Table B, Results for Method 2. Table C, Results for Method 3.
(DOC)
