# Supplementary material for: Males and Females Contribute Unequally to Offspring Genetic Diversity in the Polygynandrous Mating System of Wild Boar
Source: PLoS One. 2014 Dec 26;9(12):e115394. doi: 10.1371/journal.pone.0115394 (PMC4277350; doi:10.1371/journal.pone.0115394)
Supplement: S1 Fig — Relationship between the inferred number of reproductive individuals and the genetic diversity in paternal genotype. (DOC) [file pone.0115394.s001.doc]

Figure S1. Relationship between the inferred number of reproductive individuals and the genetic diversity in paternal genotype.


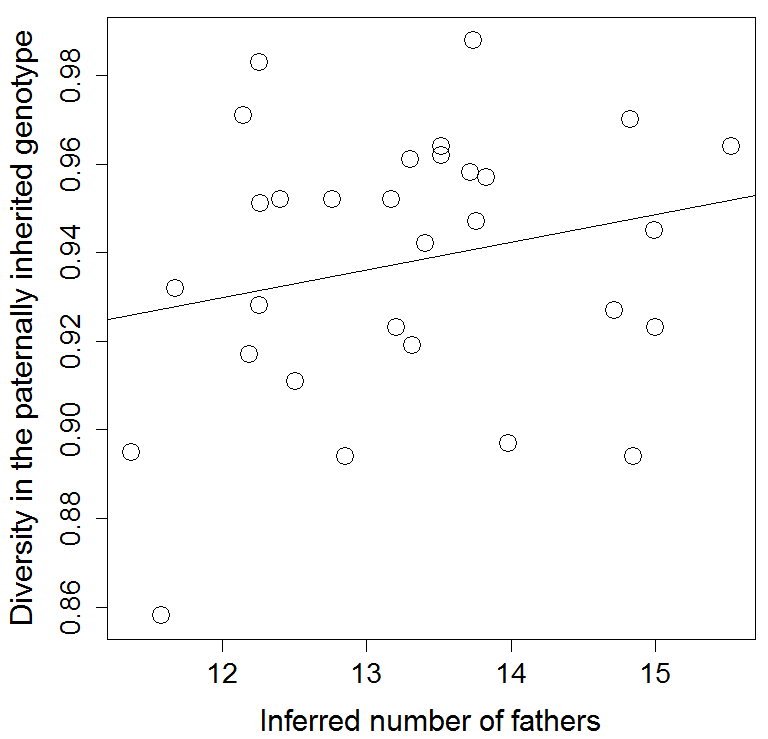


In the last analysis of wild boar data we used the genetic diversity contribution of males and females as dependent variable and number of reproductive individuals as one of the fixed factors. In the case of males, the number of reproductive individuals we used was inferred with Colony software. There could be a relationship between the inferred number of reproductive individuals and the genetic diversity in paternal genotype. Therefore, an artificial correlation between the dependent variable and the fixed factor might arise.

The following simulation procedure demonstrates that this correlation is weak and it might not invalidate the analysis.

We used the genotypes (14 microsatellite markers) of males and mothers collected in one of our wild boar populations: Santa Amalia. In this population we have 13 mothers and 16 males. We randomly mated these individuals and generated the genotypes of simulated offspring. The distribution of the litter size among females was the same as in the real population (N = 45 offspring). To simulate MP, females mated with two males (*a* and *b*). Within litters, both males were assumed to have equal probability of paternity. When the litter size was odd, the number of offspring sired by male *a* was higher. To simulate polygyny, males were allowed to mate with more than one female. Variable male reproductive success was simulated by assuming 12 males having a probability *p* of mating, 2 males with a probability 2*p* of mating and 2 males with a probability 3*p* of mating.

We simulated this system 1000 times and selected the first 30 runs in which the array of offspring were sired by 12 males. Therefore, we had 30 different sets of simulated offspring that were sired by the same number of fathers (12).

For each of these 30 sets of offspring we quantified the diversity of the paternally inherited genotypes.

On the other hand, for each of these 30 sets of offspring we estimated the number of fathers with Colony software. We included information about the mothers. However, we did not include any information about the fathers to generate variation in results (when we included in the software the genetic information of fathers, the results did not show variation: 12 fathers in all offspring sets).

Figure S1 shows the relationship between the inferred number of fathers (Colony results) and the diversity in the paternally inherited genotypes for the 30 different sets of simulated offspring. Despite the positive trend, the relationship is weak (N = 30, r^2^ = 0.052, p = 0.227) and it might not invalidate the analysis in the main text.
