# Supplementary material for: Males and Females Contribute Unequally to Offspring Genetic Diversity in the Polygynandrous Mating System of Wild Boar
Source: PLoS One. 2014 Dec 26;9(12):e115394. doi: 10.1371/journal.pone.0115394 (PMC4277350; doi:10.1371/journal.pone.0115394)
Supplement: S1 Table — Sample sizes for each hunting site. (DOC) [file pone.0115394.s002.doc]

Table S1. Sample sizes for each hunting site.

| Hunting sites | Area | Population | Nmothers | Nfoetuses | Nmales |
| --- | --- | --- | --- | --- | --- |
| Évora | Iberian Peninsula | WIP | 5 | 27 | 0 |
| Alqueva | Iberian Peninsula | WIP | 11 | 45 | 10 |
| Vila Viçosa | Iberian Peninsula | WIP | 11 | 44 | 28 |
| Azagala | Iberian Peninsula | AZA | 35 | 135 | 18 |
| Santa Amalia | Iberian Peninsula | SAM | 13 | 45 | 16 |
| Kisbajom | Hungary | HUN | 0 | 0 | 2 |
| Lábod | Hungary | HUN | 0 | 0 | 1 |
| Szulok | Hungary | HUN | 1 | 7 | 1 |
| Cserénfa | Hungary | HUN | 19 | 101 | 0 |
| Kereki | Hungary | HUN | 9 | 49 | 7 |
| Kereki Kapasi | Hungary | HUN | 1 | 10 | 1 |
| Pusztaszemes | Hungary | HUN | 3 | 20 | 3 |
| Karád | Hungary | HUN | 0 | 0 | 2 |
| Tótokilap | Hungary | HUN | 2 | 19 | 2 |
| Total |  |  | 110 | 502 | 91 |

Table shows whether the sites were located in Iberian Peninsula or Hungary and the genetically different population they belong to (WIP: Western Iberian Peninsula; AZA: Azagala, SAM: Santa Amalia; HUN: Hungary). Nmothers: number of pregnant females; Nfoetuses: number of foetuses; Nmales: size of the random sample of males.
