# Supplementary material for: Males and Females Contribute Unequally to Offspring Genetic Diversity in the Polygynandrous Mating System of Wild Boar
Source: PLoS One. 2014 Dec 26;9(12):e115394. doi: 10.1371/journal.pone.0115394 (PMC4277350; doi:10.1371/journal.pone.0115394)
Supplement: S2 Table — Sample sizes and heterozygosity for each genetically different population. (DOC) [file pone.0115394.s003.doc]

Table S2. Sample sizes and heterozygosity for each genetically different population.

| Population | Nmothers | Nfoetuses | Nmales | Ho | He |  |
| --- | --- | --- | --- | --- | --- | --- |
| WIP | 27 | 116 | 38 | 0.548 | 0.553 |  |
| AZA | 35 | 135 | 18 | 0.658 | 0.632 |  |
| SAM | 13 | 45 | 16 | 0.634 | 0.632 |  |
| HUN | 35 | 206 | 19 | 0.683 | 0.692 |  |
| Total | 110 | 502 | 91 | 0.631 | 0.627 | Overall |

Nmothers: number of pregnant females; Nfoetuses: number of foetuses; Nmales: size of the random sample of males. Table also shows observed (Ho) and expected (He) heterozygosities (Belkhir et al. 2004).

Reference:

Belkhir K., Borsa P., Chikhi L., Raufaste N. & Bonhomme F. 2004 GENETIX 4.05, logiciel sous Windows TM pour la génétique des populations. Laboratoire Génome, Populations, Interactions, CNRS UMR 5000, Université de Montpellier II, Montpellier (France).
