# Supplementary material for: Males and Females Contribute Unequally to Offspring Genetic Diversity in the Polygynandrous Mating System of Wild Boar
Source: PLoS One. 2014 Dec 26;9(12):e115394. doi: 10.1371/journal.pone.0115394 (PMC4277350; doi:10.1371/journal.pone.0115394)
Supplement: S3 Table — Genetic information of microsatellite markers. (DOC) [file pone.0115394.s004.doc]

Table S3. Genetic information of microsatellite markers.

| Marker | Range | A | Ho | He |
| --- | --- | --- | --- | --- |
| Sw24 | 111-139 | 10 | 0.688 | 0.631 |
| S0155 | 165-183 | 9 | 0.532 | 0.564 |
| Sw936 | 111-131 | 9 | 0.642 | 0.688 |
| Sw2410 | 122-142 | 10 | 0.650 | 0.675 |
| S0005 | 223-277 | 24 | 0.833 | 0.837 |
| Sw632 | 177-197 | 9 | 0.702 | 0.664 |
| Sw857 | 170-178 | 5 | 0.451 | 0.412 |
| S0226 | 202-204 | 6 | 0.486 | 0.502 |
| Sw72 | 119-133 | 7 | 0.705 | 0.662 |
| Sw240 | 112-130 | 8 | 0.757 | 0.659 |
| S0068 | 246-280 | 14 | 0.825 | 0.812 |
| S0101 | 216-236 | 10 | 0.524 | 0.561 |
| Sw122 | 119-143 | 9 | 0.543 | 0.583 |
| Sw2008 | 116-132 | 5 | 0.500 | 0.523 |

Table shows allelic range, mean number of alleles (A) and observed (Ho) and expected (He) heterozygosities for each microsatellite marker (Belkhir et al. 2004).

Reference:

Belkhir K., Borsa P., Chikhi L., Raufaste N. & Bonhomme F. 2004 GENETIX 4.05, logiciel sous Windows TM pour la génétique des populations. Laboratoire Génome, Populations, Interactions, CNRS UMR 5000, Université de Montpellier II, Montpellier (France).
