# Supplementary material for: Males and Females Contribute Unequally to Offspring Genetic Diversity in the Polygynandrous Mating System of Wild Boar
Source: PLoS One. 2014 Dec 26;9(12):e115394. doi: 10.1371/journal.pone.0115394 (PMC4277350; doi:10.1371/journal.pone.0115394)
Supplement: S1 File — The study area and the places in which hunting events were conducted. This file also contains Figures A and B. Figure A, Iberian Peninsula. Figure B, Hungary. (DOC) [file pone.0115394.s005.doc]

File S1. The study area and the places in which hunting events were conducted. Figure A. Iberian Peninsula. Figure B. Hungary.
