# Supplementary material for: Males and Females Contribute Unequally to Offspring Genetic Diversity in the Polygynandrous Mating System of Wild Boar
Source: PLoS One. 2014 Dec 26;9(12):e115394. doi: 10.1371/journal.pone.0115394 (PMC4277350; doi:10.1371/journal.pone.0115394)
Supplement: S2 File — Structure results after splitting Iberian Peninsula and Hungary. This file also contains Figure S2. Figure S2a, Log-likelihood values for Iberian Peninsula (ten independent runs). Figure S2b, Membership coefficient of probability for Iberian Peninsula (K = 3). Figure S2c, Log-likelihood values for Hungary (ten independent runs). (DOC) [file pone.0115394.s006.doc]

File S2. Structure results after splitting Iberian Peninsula and Hungary. Figure S2a. Log-likelihood values for Iberian Peninsula (ten independent runs). Figure S2b. Membership coefficient of probability for Iberian Peninsula (*K* = 3). Figure S2c. Log-likelihood values for Hungary (ten independent runs).

For Iberian Peninsula, STRUCTURE results show that *K* = 3 was the most probable number of genetic clusters in our data set (Figure S2a). Despite the existence of some migrant or descendant of migrant individuals, the genetic clusters generally matched with three geographic areas: western Iberian Peninsula, Azagala and Santa Amalia (Figure S2b).

For Hungary, STRUCTURE results show that *K* = 1 was the most probable number of genetic clusters in our data set (Figure S2c).
