# Supplementary material for: Males and Females Contribute Unequally to Offspring Genetic Diversity in the Polygynandrous Mating System of Wild Boar
Source: PLoS One. 2014 Dec 26;9(12):e115394. doi: 10.1371/journal.pone.0115394 (PMC4277350; doi:10.1371/journal.pone.0115394)
Supplement: S3 File — Relationship between genetic diversity in parental genotypes and covariates. This file also contains Figure S3. Figure S3a, Covariate: Number of reproductive individuals. Figure S3b, Covariate: Genetic diversity in adults. (DOC) [file pone.0115394.s007.doc]

File S3. Relationship between genetic diversity in parental genotypes and covariates. Figure S3a. Covariate: Number of reproductive individuals. Figure S3b. Covariate: Genetic diversity in adults.

Graphs show observed values of genetic diversity in parental genotypes by using Method 1. For methods 2 and 3 graphs are similar. Data are split in those for males (grey points and grey line) and for females (black points and black line).
