# Supplementary material for: Males and Females Contribute Unequally to Offspring Genetic Diversity in the Polygynandrous Mating System of Wild Boar
Source: PLoS One. 2014 Dec 26;9(12):e115394. doi: 10.1371/journal.pone.0115394 (PMC4277350; doi:10.1371/journal.pone.0115394)
Supplement: S4 File — Results after using MOL_COANC for paternity analyses. This file also contains Tables A–C. Table A, Results for Method 1. Table B, Results for Method 2. Table C, Results for Method 3. (DOC) [file pone.0115394.s008.doc]

File S4: Results after using MOL_COANC in paternity analyses. Table A. Results for Method 1. Table B. Results for Method 2. Table C. Results for Method 3.

| A ) Method 1 | post. mean | Lower 95% CI | Upper 95% CI | P_MCMC_ |
| --- | --- | --- | --- | --- |
| Intercept | -0.242 | -0.368 | -0.095 | <0.001 |
| Genetic diversity in adults | 0.957 | 0.870 | 1.031 | <0.001 |
| Number of reproductive individuals | 0.005 | 0.003 | 0.009 | <0.001 |
| Sex | 0.226 | 0.158 | 0.297 | <0.001 |
| B) Method 2 | post. mean | Lower 95% CI | Upper 95% CI | P_MCMC_ |
| Intercept | -0.078 | -0.196 | 0.031 | 0.184 |
| Genetic diversity in adults | 0.924 | 0.855 | 0.997 | <0.001 |
| Number of reproductive individuals | 0.005 | 0.003 | 0.007 | 0.002 |
| Sex | 0.084 | 0.027 | 0.138 | 0.002 |
| C) Method 3 | num. d.f. | den. d.f. | F | P |
| Intercept | -0.196 | 0.320 | -0.077 | <0.001 |
| Genetic diversity in adults | 0.993 | 0.860 | 1.012 | <0.001 |
| Number of reproductive individuals | 0.005 | 0.002 | 0.008 | 0.002 |
| Sex | 0.292 | 0.218 | 0.358 | <0.001 |

Fixed effects of a LMM fitted using MCMC in which we compared the genetic diversity of paternally and maternally transmitted genotypes after controlling for the genetic diversity in adults and the number of reproductive individuals. The table shows the posterior estimate of the effects, the 95% credibility interval and the probability that the null hypothesis is true (effect = 0). We accepted families with incompatibilities in one locus.

The number of reproductive males obtained was 20 in Portugal, 25 in Azagala, 9 in Santa Amalia and 48 in Hungary. MOL_COANC inferred lower numbers of reproductive males than those with COLONY. In this case, only the Hungarian population had a bigger number of reproductive males than reproductive females.

Despite the different number of reproductive males, the main finding of the main text remained: after controlling for the genetic diversity in adults and the number of reproductive individuals, paternal half genotypes of foetuses remained more genetically diverse than maternal half genotypes. When we used MOL_COANC the differences were even higher.
